# Comparative Studies of Copy Number Variation Detection Methods for Next-Generation Sequencing Technologies

**DOI:** 10.1371/journal.pone.0059128

**Published:** 2013-03-20

**Authors:** Junbo Duan, Ji-Gang Zhang, Hong-Wen Deng, Yu-Ping Wang

**Affiliations:** 1 Department of Biomedical Engineering, Tulane University, New Orleans, Louisiana, United States of America; 2 Department of Biostatistics and Bioinformatics, Tulane University, New Orleans, Louisiana, United States of America; 3 Center for Bioinformatics and Genomics, Tulane University, New Orleans, Louisiana, United States of America; University of Lausanne, Switzerland

## Abstract

Copy number variation (CNV) has played an important role in studies of susceptibility or resistance to complex diseases. Traditional methods such as fluorescence *in situ* hybridization (FISH) and array comparative genomic hybridization (aCGH) suffer from low resolution of genomic regions. Following the emergence of next generation sequencing (NGS) technologies, CNV detection methods based on the short read data have recently been developed. However, due to the relatively young age of the procedures, their performance is not fully understood. To help investigators choose suitable methods to detect CNVs, comparative studies are needed. We compared six publicly available CNV detection methods: CNV-seq, FREEC, readDepth, CNVnator, SegSeq and event-wise testing (EWT). They are evaluated both on simulated and real data with different experiment settings. The receiver operating characteristic (ROC) curve is employed to demonstrate the detection performance in terms of sensitivity and specificity, box plot is employed to compare their performances in terms of breakpoint and copy number estimation, Venn diagram is employed to show the consistency among these methods, and *F*-score is employed to show the overlapping quality of detected CNVs. The computational demands are also studied. The results of our work provide a comprehensive evaluation on the performances of the selected CNV detection methods, which will help biological investigators choose the best possible method.

## Introduction

Copy number variation (CNV) [1] is a form of structural variation (SV) [2], [3] in the genome. Usually, CNV refers to the duplication or deletion of DNA segments larger than 1 kbp [4]. Iafrate *et al.* [5] showed that CNVs are present in human populations with high frequency (more than 10 percent). [6] showed that in an individual genome the average size of CNVs is 3.5±0.5 Mbp (0.1 percent). Many studies have shown that CNVs are associated with complex diseases such as autism [7], schizophrenia [8], Alzheimer disease [9], cancer [10], *etc.*

Traditionally, fluorescence in situ hybridization (FISH) and array comparative genomic hybridization (aCGH) are employed to detect CNVs. However, because of their low resolutions (about 5∼10 Mbp for FISH, and 10∼25 kbp with 1 million probes for aCGH [11]), short CNVs are still difficult to detect. In the last few years, the NGS technology brought revolutionary breakthroughs and is used in various fields of life science [12], *e.g.*, for the detection of CNVs with high resolution (<10 kbp) [13]. Recently a variety of CNV detection methods were proposed [11], [14]–[27] (see Table 1). Motivated by a comparative study of CNV detection methods based on aCGH technique [28], we conducted a comprehensive comparison of six representative CNV detection methods based on NGS under different sets of conditions. We hope this study can provide researchers with a clear understanding of the method’s strengths and weaknesses, and use this information to choose suitable methods in practice.

**Table 1:** List of selected CNV detection methods.

Method	Reference	Language	Control required?	Input format	GC correction	single-end/pair-end	Methodology characteristics
CNV-seq	[15]	R, perl	Yes	hits	No	single-end	statistical testing
FREEC	[21]	C	Optional	SAM,BAM,bed,*etc.*	Optional	both	LASSO regression
readDepth	[22]	R	No	bed	Yes	both	CBS, LOESS regression
CNVnator	[23]	C	No	BAM	Yes	both	mean shift algorithm
SegSeq	[14]	Matlab	Yes	bed	No	single-end	statistical testing,CBS
EWT (RDXplorer)	[11]	R, python	No	BAM	Yes	single-end	statistical testing
cnD	[16]	D	No	SAM,BAM	No	both	HMM, Viterbi algorithm
CNVer	[17]	C	No	BAM	Yes	pair-end	maximum-likelihood, graphic flow
CopySeq	[18]	Java	No	BAM	Yes	pair-end	MAP estimator
rSW-seq	[19]	NA	Yes	NA	Yes	single-end	Smith-Waterman algorithm
CNAseg	[20]	R	Yes	BAM	No	pair-end	wavelet transform and HMM
CNAnorm	[24]	R	Yes	SAM,BAM	Yes	both	linear regression or CBS
cn.MOPS	[26]	R, C++	multiple samples	BAM or data matrix	No	both	mixture of Poissons, MAP, EM, CBS
JointSLM	[27]	R, Fortran	multiple samples	data matrix	Yes	both	HMM, ML estimator, Viterbi algorithm

There are two main categories of NGS based CNV-detection methods: the pair-end mapping (PEM) based and the depth of coverage (DOC) based method [29]. PEM based methods are suitable to detect balanced SVs such as inversions and CNVs with small size. The DOC methods are more popularly employed by most CNV detection tools.

DOC based methods first pile up the aligned reads at the genomic coordinate, and then calculate the read counts across sliding [15] or non-overlapping windows (or bins) [11], [14], [21], [23], yielding the so-called read depth signal. In the case of CNV-seq [15] and SegSeq [14], the ratios of the read counts do not require further normalization due to the requirement of a control sample [19]. Otherwise, normalization such as GC-content [11], [23] and mapability [22] correction is required. The normalized read depth signal is processed following one of the two ways: (1) first segmented by local change-point (or segmentation, partition) algorithms and a merge procedure [14] (*e.g.* readDepth [22] uses circular binary segmentation (CBS); CNVnator [23] uses mean shift; and FREEC [21] uses Lasso based method); (2) tested by a statistical hypothesis at each window (*e.g.* event-wise testing (EWT) [11]) or several consecutive windows [15].

A CNV is characterized by the break point loci (starting and ending points), single copy length and number of copies. Precise break point loci and copy number estimation are always desirable. Normally, shorter CNVs are more difficult to detect than longer ones. Also, it is easier to detect copy number greatly deviated CNVs than slightly deviated ones. (*e.g.* a homozygous deletion is easier to detect than a heterozygous deletion). In addition, it is known that higher coverage can provide higher resolution of break point detection, yielding higher accuracy. Therefore, these parameters are important factors to be studied.

Based on the results of the comparative studies, the tested methods were ranked in terms of break point detection, copy number estimation, false positive rate, true positive rate, computation time and peak memory usage, and guidelines for the selection of appropriate methods under a specific set of conditions were given. Moreover, the advantages and disadvantages of each method, the related issues of CNV detection from NGS data, as well as the directions for further improving current methods and software were discussed.

## Methods

### Copy Number Detection Methods Used in the Studies

A number of CNV detection methods have been published recently for NGS data analysis [11], [14]–[27] (see Table 1). These methods differ in statistical model, parameter, methodology, programming language, operating system, input requirement, output format, and signature used. Based on these factors, as well as the public availability, implementation stability, and the citation in literature, six popular and representative methods were selected: CNV-seq, FREEC, readDepth, CNVnator, SegSeq, and event-wise testing (EWT). These methods are no means exclusive, but we believe they represent a fair number of CNV detection methods for NGS data. Table 1 lists the detailed information of the existing CNV detection methods to the best of the authors’ knowledge. rSW-seq was excluded because of no publicly available codes. CNAnorm was not available when we started our study. cnD and CNAseg were excluded because of stability issue. From the same dataset, there were issues getting the code to fully perform to our expectations. Our study focused on the method with DOC signature, so CNVer and CopySeq were excluded because they combine both ROC and PEM signature. A comparison between the combined signature based methods with the single signature based methods can be found in [17]. It was already shown that modeling across samples can improve the performance considerably [26], [27], and therefore JointSLM and cn.MOPS were not compared in this paper for fairness. Since they are developed for the purpose of population studies, multiple samples (recommended at least 10 and 6 samples respectively) are required.

### Simulated Data Processing

**Figure 1:**
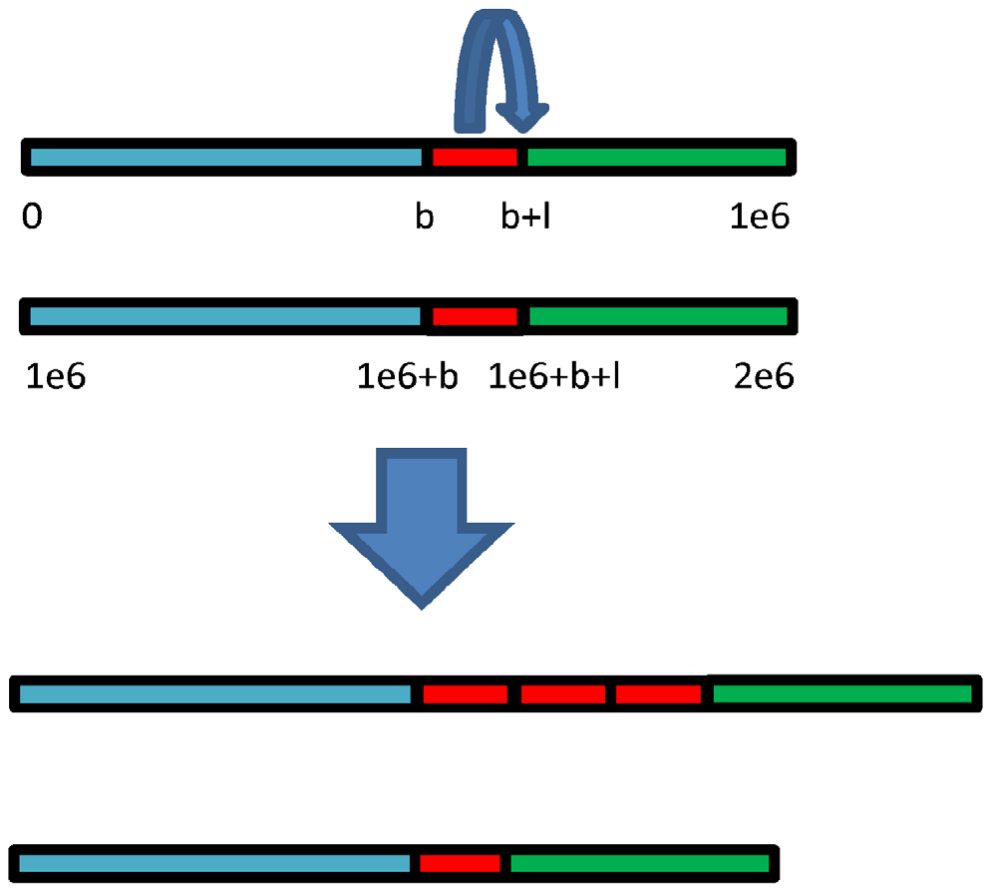
Schematic demonstration of the generation of the test genome (the lower one) from the control genome (the upper one) when copy number *n* = 4. A DNA segment of length *l* bp (the length of a single red block) starting from locus *b* is copied and inserted *n*−2 times.

A simulation study was carried out to compare the performance of each CNV detection method. In such a case, the parameters of CNVs to be estimated were known in advance as the ground truth. Figure 1 shows the simulation.

The following parameters concerning CNVs are mainly under our studies because of their biological significance:

- Single copy length (*l*): the length of a single copy. As is shown in Figure 1, it is the length of a single red block.
- Copy number (*n*): the number of duplicated red blocks in Figure 1. For diploid genome, suppose *n* = 2 as normal, *n* = 0, 1 as loss, and *n*>2 as gain.
- Coverage (*c*): the average number of reads that cover each base pair in the genome. It can be calculated from the length of the genome *G*, the number of short reads *N*, and the average read length *L* as 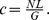

The aforementioned parameters were tuned during the simulation. The read length *L* was fixed to 36 bp to be consistent with the Illumina platform; the genome length *G* was fixed to 1 Mbp; and the locus of break point (where a CNV starts) was fixed to *b*. In the studies, the maximum single copy length was constrained to be within 6 kbp, because our studies showed that CNVs with size larger than 6 kbp can be detected by all methods. For the same reason, the maximum copy number was constrained to be within 6.

For each different combination of parameters (*l, n, c*) (see Table 2), 1000 Monte Carlo trials were carried out. For each trial, the procedure of simulating and detecting CNVs was as follows:

**Table 2:** List of combinations of parameters (*l, n, c*) in simulation.

Parameter of interest	Values	Fixed parameters
*l* (kbp)	0.8,1,2,6	*n* = 6,*c* = 5
*n*	0,1,3,6	*l* = 6 kbp, *c* = 5
*c*	3,5,10,30	*l* = 6 kbp, *n* = 5

1. Generation of the reference and control genome. First, all the known CNVs (CVNs listed in database of genomic variants (DGV)) of chromosome 1 and 21 of NCBI36/hg18 were removed. Then ten sequences of length 1 Mbp were extracted (9 from chromosome 1 and 1 from chromosome 21). These 1 Mbp sequences were used randomly as the reference genome, and a diploid control genome was generated from this reference.
2. Generation of the test genome. The test genome was generated from the control genome by introducing CNVs with given parameters. For *n*>2, a section staring from the predefined break point *b* with length *l* was copied, and inserted *n*−2 times into the genomic locus *b+l* (see Figure 1). So in the genomic locus between *b* and *b+l*, a gain with copy number *n* was expected to be observed. For *n* <2, 2 − *n* copy(s) was (were) deleted.
3. Introduction of SNPs and indels. The frequency is 5 SNPs/kbp and 0.5 indels/kbp, and the indels have random length of 1∼3 bp.
4. Generation of reads. To simulate the non-uniform bias, each locus was assigned with a sample probability *p*, which is the product of mapability and GC-content profile downloaded from the readDepth’s website. From both the control and test genome, reads were sampled randomly according to the probability *p* on the whole genomic loci. For fixed coverage *c*, the number of short reads *N* can be calculated as 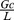.
5. Alignment of reads to the reference. The mapping tool Bowtie [30] was used to map all the reads to the reference genome with default options, yielding output SAM file. Then the SAMtools [31] was used to convert SAM to BAM file, and generate hits file and bed file from BAM file.
6. Run of each CNV detection method. The configuration of parameters is explained in the discussion section.
7. Summarization of the outputs of each method. The results of each method were sorted according to the following information: parameters used, number of Monte Carlo trial, starting break point locus, length of CNV, and the copy number.

### Real Data Processing

To compare the performances of the CNV detection methods on the real data, a BAM file of the chromosome 21 of NA19240 (Yoruba female) was downloaded from the website of Illumina. This BAM file contains approximately 14.7 million reads, which were aligned to the NCBI36/hg18 reference genome. The coverage is 11, indicating a medium coverage. For the methods that require a control sample (*i.e.* CNV-seq and SegSeq), we generated the control sequencing data from chromosome 21 of NCBI36/hg18 reference with the same sequencing parameters (coverage and average read length).

The detected CNVs by aforementioned six methods were compared with those retrieved from the database of genomic variants (DGV), which lists all the discovered CNVs reported in the literature. The option of filter query was ‘external sample id = NA19240, chromosome = 21, assembly = NCBI36/hg18, variant type = CNV’.

To compare the computational performance of CNV detection methods, a high coverage data set is required. So from the website of the 1000 Genomes Project, a BAM file of chromosome 1 of NA19240 was thus downloaded, which has 0.22 billion short reads with the coverage of 34.

### Performance Evaluation Criteria

Since it is difficult to compare the performances of CNV detection methods from an algorithmic point of view, the black-box testing method was employed, which is widely used in the software engineering. Without knowing the explicit structure, the black-box testing can compare the relative performances of multiple software tools by analyzing their inputs and outputs. To help the researchers have an overview of the performance of each tested method in terms of both break point detection and copy number estimation, we showed the estimates by the box plots. Furthermore, we listed the means and standard deviations of the estimates errors. The detection performances were evaluated by the receiver operating characteristic (ROC) curves, the precision- recall curves and the *F*-scores. In the real data processing, the Venn diagram was used to show the consistency of the detected CNVs using each individual method, and the *F*-score was employed to quantitatively evaluate the quality of detected CNVs. Computational demands are also important factors for analyzing huge amount of NGS data, so both the computation time and peak memory usage were investigated.

For the simulated data processing, we followed the comparative study of array CGH analysis tools [28], where the receiver operating characteristic (ROC) curve was used to evaluate the detection of the performance. The ROC curve is a graphical plot of the true positive rate (TPR, equivalent to sensitivity or recall) *vs* false positive rate (FPR, equivalent to 1-specificity). These two measurements are closely related to the type I and type II error. The ideal detection is expected to achieve TPR = 1 and FPR = 0. However, in real world these two measurements are always in contradiction: when one wants to improve the TPR, the FPR will degenerate at the same time, and *vice versa*. So usually one has to decide a trade-off between these two measurements, which depends on the study design. For example, in the biomarker identification studies, the researchers want to identity biomarkers that could be associated with a particular disease. In this case, high sensitivity is preferable since false detections can be further removed by the experimental validation. A point in the ROC curve that is closer to the northwest corner (*i.e.* with greater TPR and lower FPR) is believed to indicate better performance than the one further away. In each Monte Carlo trial, the detected CNV was compared with the ground truth, yielding a point on the ROC plot. The TPR and FPR were calculated in the unit of base pair. The true positive rate was calculated as the ratio between the number of base pairs in detected CNVs that overlap with the ground truth, and the number of base pairs in the ground truth; the false positive rate was calculated as the ratio between the number of base pairs in detected CNVs that do no overlap with the ground truth, and the number of base pairs not in the ground truth.

The box plot was employed to depict the estimation performance of both the break point position and the copy number. The box plot depicts the minimum, the lower quartile, the median, the upper quartile and the maximum of the estimates for the 1000 Monte Carlo trials. So from the box plot, the distribution of the estimates precision is clearly presented.

To evaluate the overlap quality, the *F*-score [2] was introduced, which takes value ranging from 0 to 1. A lower score indicates a poor consistency with the ground truth while a higher score indicates a better consistency. To calculate the *F*-score for each detected CNV, the following is considered: if it has no overlap with any ground truth (CNVs in DGV), the *F*-score is set to be 0; otherwise, 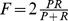, where *P* is the precision (percent of the detected CNV that overlaps with the ground truth) and *R* is the recall (percent of the ground truth that overlaps with the detected CNV) [2]. The Venn diagram was used to demonstrate the consistency between detected CNVs with different methods. In order to take into account of the lengths of CNVs, the genome was segmented into blocks of the same length (100 bp), and the overlaps were counted block by block.

The computational demand is an important issue in real data processing. We consider both the computation time and memory usage as the evaluation criteria. In particular, readDepth [22] supports the multi-core computation, so this option was enabled to achieve its best computation speed. Note that other methods may also support the acceleration feature, depending on whether the programming language supports the multi-core and parallelizing computation or not. Memory usage is the peak memory occupied during the execution, including the related software, *e.g.* Matlab.

## Results

### Simulation Studies

The experiments were run in terms of single copy length, copy number and coverage.

#### Single copy length

**Figure 2:**
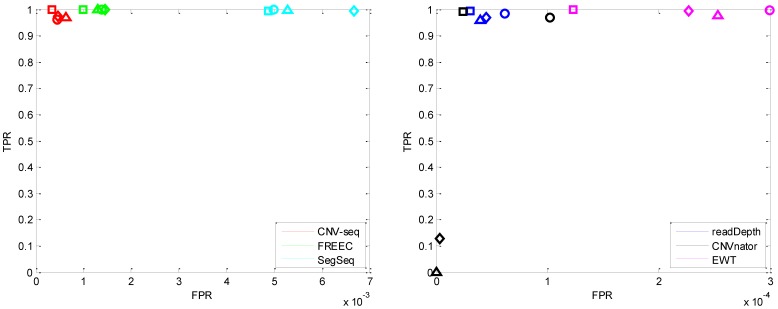
The ROC curves with different single copy length: 0.8 kbp (triangle), 1 kbp (diamond), 2 kbp (circle) and 6 kbp (square). The coverage is fixed to 5 and copy number is fixed to 6. Notice that the horizontal axes have different scales.

**Figure 3:**
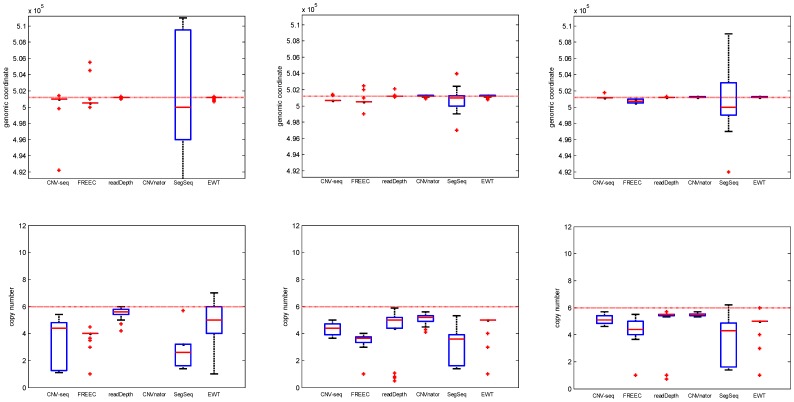
The box plot of the break point position estimates (first row) and copy number estimates (second row) of CNVs with different single copy length: 0.8 kbp, 2 kbp and 6 kbp, respectively. The coverage is fixed to 5 and the copy number is fixed to 6. The horizontal red dotted lines indicate the ground truth values; the red solid lines indicate the mean values; and the red pluses indicate the outliers.

The performances for different single copy lengths (*l* = 0.8, 1, 2 and 6 kbp) are shown in Figure 2, 3, S1 and Table S1. The coverage *c* is fixed to 5, and the copy number *n* is fixed to 6 (see Table 2).

In Figure 2, each point of the ROC curve is the average of 1000 points, each corresponding to a Monte Carlo trial with the same parameters. It is shown that with an increase of single copy length, the true positive detection rate increases. This observation is consistent with our expectation, because CNVs of large sizes are easy to detect. CNVnator fails when the single copy length is lower than 2 kbp. readDepth, CNVnator and EWT achieve lower false positive rate (at the amplitude of 10^−4^) compared with CNV-seq, FREEC and SegSeq (at the amplitude of 10^−3^). Here it is necessary to note that the former three methods do not require a control sample, but CNV-seq and SegSeq do (see Table 1).

Figure 3 shows that with increases of the single copy length, the estimates of readDepth are stable. readDepth and EWT achieve the best performance on break point position estimation, while CNVnator and readDepth achieve the best performance on copy number estimation. The means and standard deviations of estimation error of break point position and copy number are listed in Table 3 and 4 respectively.

**Table 3:** The means and standard deviations of estimation error of break point position.

	CNV-seq	FREEC	readDepth	CNVnator	SegSeq	EWT
*l* = 0.8 kbp, *n* = 6, *c* = 5	395±1.67e3	729±87	32±10	NA	686±1.78e3	36±17
*l* = 2 kbp, *n* = 6, *c* = 5	449±218	793±752	29±92	2±91	884±552	83±82
*l* = 6 kbp, *n* = 6, *c* = 5	100±138	479±251	28±22	2±48	1.22e3±1.37e3	31±14
*l* = 6 kbp, *n* = 0, *c* = 5	116±270	359±548	33±0	113±51	417±9.07e3	5.13e3±5.05e3
*l* = 6 kbp, *n* = 1, *c* = 5	613±838	274±136	25±80	177±442	194±5.28e3	30±194
*l* = 6 kbp, *n* = 3, *c* = 5	1.28e3±1.57e3	269±178	1.6e3±2.15e3	79±404	561±1.09e3	1.71e3±2.05e3
*l* = 6 kbp, *n* = 6, *c* = 3	643±458	669±183	NA	49±363	2.76e3±1.48e3	75±88
*l* = 6 kbp, *n* = 6, *c* = 10	203±81	729±50	32±10	59±290	88±9.15e3	36±17
*l* = 6 kbp, *n* = 6, *c* = 30	24±28	734±0	48±426	12±41	477±3.32e3	33±0

**Table 4:** The means and standard deviations of estimation error of copy number.

	CNV-seq	FREEC	readDepth	CNVnator	SegSeq	EWT
*l* = 0.8 kbp, *n* = 6, *c* = 5	2.44±1.46	2.53±0.26	0.89±0.68	NA	3.46±1.08	1.38±0.93
*l* = 2 kbp, *n* = 6, *c* = 5	1.61±0.22	2.3±0.51	1.23±1.08	0.85±0.22	2.86±1.07	1.30±0.79
*l* = 6 kbp, *n* = 6, *c* = 5	0.87±0.19	1.56±0.53	0.82±0.78	0.50±0.08	2.46±1.56	1.10±0.93
*l* = 6 kbp, *n* = 0, *c* = 5	0.15±0.06	1.12±1.41	0.06±0.01	0.02±0.01	1.73±0.70	1.27±2.08
*l* = 6 kbp, *n* = 1, *c* = 5	0.14±0.05	0.15±0.53	0.02±0.13	0.17±0.05	0.49±0.46	0.36±0.89
*l* = 6 kbp, *n* = 3, *c* = 5	0.21±0.08	0.00±0.00	0.46±0.99	0.12±0.08	0.63±0.59	0.03±0.30
*l* = 6 kbp, *n* = 6, *c* = 3	1.12±0.23	1.76±0.48	NA	0.45±0.14	2.43±1.33	1.13±0.82
*l* = 6 kbp, *n* = 6, *c* = 10	0.74±0.14	1.69±0.32	0.66±0.45	0.49±0.07	3.27±1.47	0.89±0.96
*l* = 6 kbp, *n* = 6, *c* = 30	0.73±0.08	1.96±0.30	0.84±0.57	0.70±0.04	4.10±0.69	1.24±0.90

#### Copy number

**Figure 4:**
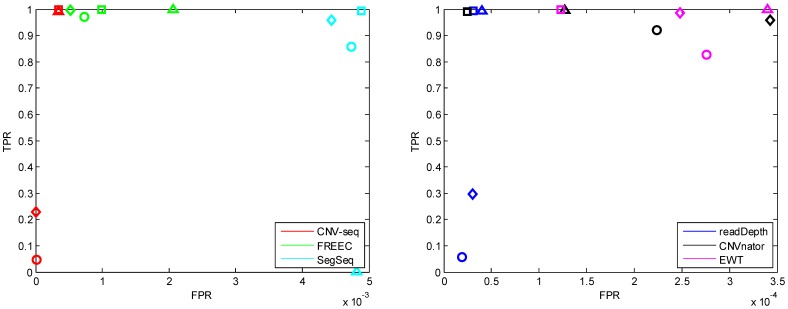
The ROC curves with different copy number: 0 (triangle), 1 (diamond), 3 (circle) and 6 (square). The coverage is fixed to 5 and single copy length is fixed to 6 kbp.

**Figure 5:**
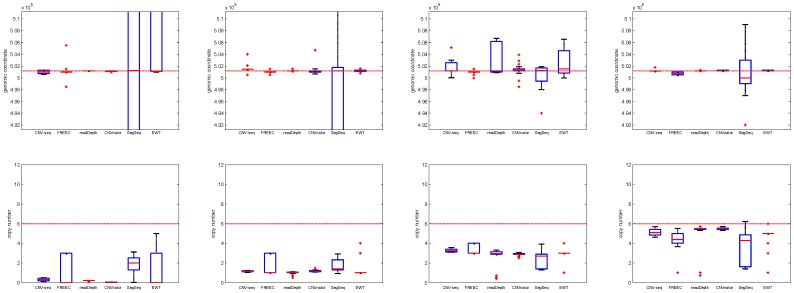
The box plots of the break point position estimates (first row) and copy number estimates (second row) of CNVs with different copy number: 0, 1, 3 and 6, respectively. The coverage is fixed to 5 and single copy length is fixed to 6 kbp. The horizontal red dotted lines indicate the ground truth values; the red solid lines indicate the mean value; and the red pluses indicate outliers.

The performances for different copy numbers (0, 1, 3 and 6) are shown in Figure 4, 5 and S2. The means and standard deviations of the estimation error for break point position and copy number are listed in Table 3 and 4 respectively. The coverage is fixed to 5, and the single copy length is fixed to 6 kbp (see Table 2).

We observe that (Figure 4) all the methods except SegSeq have the lowest TPR at copy number 3. Here we note that copy number 1 indicates a single copy loss, while copy number 3 indicates a single copy gain. It is more difficult to detect CNVs in these two cases than in the case when copy number is 0 or 6. CNV-seq and readDepth fail (TPR is very low) at copy number 1 and 3; SegSeq fails at copy number 0. It is shown that (box plots in Figure 5) the break point estimate and copy number estimate of CNVnator are the best compared with other methods, and the estimates do not vary much with respect to the change of copy number.

Because of the presence of SNPs and indels in the test genome, around 20% reads cannot be aligned. This affects the read depth greatly. So for *n* <2 the copy number estimates tend to be overestimated (in Figure 5, the first two box plots in the lower row are above the dotted red lines), while for *n*>2, they are underestimated (the last two).

#### Coverage

**Figure 6:**
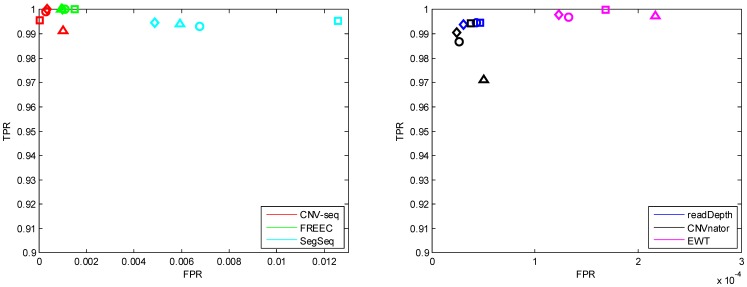
The ROC curves with different coverage: 3 (triangle), 5 (diamond), 10 (circle), 30 (square). The copy number is fixed to 6 and single copy length is fixed to 6 kbp.

**Figure 7:**
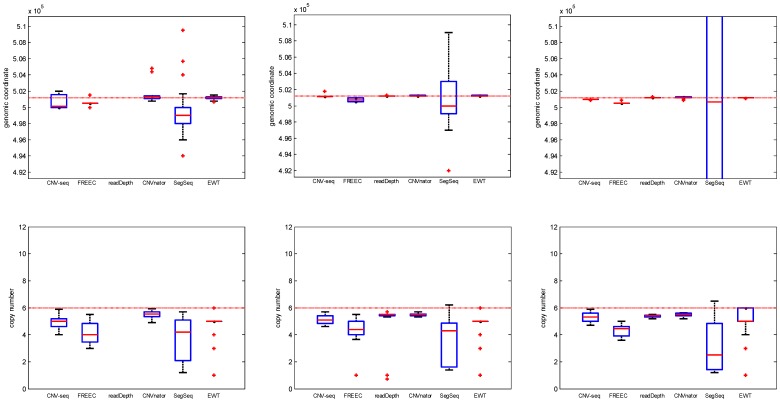
The box plots of the break point position estimates (first row) and copy number estimates (second row) of CNVs with coverage: 3, 5 and 10, respectively. The single copy length is fixed to 6 kbp and copy number is fixed to 6. The horizontal red dotted lines indicate the ground truth values; the red solid lines indicate the mean value; and the red pluses indicate outliers.

The performances of the six methods for different coverages (3, 5, 10 and 30) are shown in Figure 6, 7 and S3. It can be seen that (Figure 6) the FPR of EWT decreases (while the FPR of SegSeq increases) greatly when the coverage increases. At coverage 3, readDepth fails. It can be seen that (Figure 7) the performances (both break point position estimation and copy number estimation) of SegSeq degrade. Under investigation trial by trial, we found that when coverage increases, SegSeq tends to detect small CNV segments with random locations. So the standard deviation of break point estimates increases. These small CNV segments are of copy number around 2, so the mean of copy number estimates decreases to 2. But we can notice that the mean of break point position estimates (red solid line) approaches to the ground truth.

#### Overall performances on simulation

Table 3 and 4 list the means and standard deviations of the estimate errors of both break point position and copy number. We multiply the mean with the standard deviation of the estimate error, and take the median of the product as the ranking measurements of the break point position and copy number quality. Table S2 and S3 list the *p*-values of the pairwise Wilcoxon rank-sum test of the estimates, which show that there is no significant difference of the break point position estimates between EWT and readDepth. For FPR and TPR, we also used the median as the ranking measurements. Their performances are ranked as follows (> and = means better and no significant difference.):

- break point position estimation: readDepth = EWT>CNVnator>FREEC>CNV-seq>SegSeq;
- copy number estimation: CNVnator>CNV-seq>readDepth>FREEC>EWT>SegSeq;
- FPR: readDepth>CNVnator>EWT>CNV-seq>FREEC>SegSeq;
- TPR: FREEC>EWT>SegSeq>CNV-seq>readDepth>CNVnator.

### Real Data Studies

**Figure 8:**
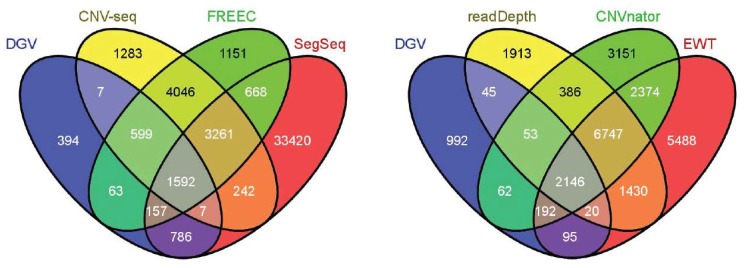
The Venn diagrams of selected CNV detection methods in the real data processing.

The results of the methods when applied to real data analysis are shown in Figure 8. The NCBI36/hg18 chromosome 21 was segmented into blocks of the same length (100 bp), and the overlaps between CNVs were analyzed block by block. Since the chromosome 21 is of length 4.7e7 bp, there are totally 4.7e5 blocks. From the left panel, it is shown that there are 1592 out of 4.7e5 blocks that are detected as CNVs by all the three methods (CNV-seq, FREEC and SegSeq), and are consistent with DGV. There are 3261 blocks that are detected as CNVs by all the three methods, but are not consistent with DGV. There are 33420 blocks that are detected only by SegSeq, indicating that SegSeq achieves higher FPR compared with CNV-seq (1283 blocks) and FREEC (1151 blocks). This is consistent with the conclusion based on the simulation.

Since readDepth, CNVnator and EWT do not require a control sample, their Venn diagrams are displayed separately in the right panel of Figure 8. There are 2146 blocks that are detected as CNVs by all the three methods, and are consistent with DGV. However, there are 6747 blocks that are detected as CNVs by all the three methods, but are not consistent with DGV.

A 7-way (selected six methods plus DGV) Venn diagram analysis was also performed. However, because this diagram is too complicated, Table 5 only presents the domains of the 7-way Venn diagram with block number greater than 1000. For example, the third column means there are 1235 blocks that are detected as CNVs by CNVnator and EWT, but considered as normal by the rest methods and the DGV. It shows that there are 2705 blocks that are detected by all six methods, and are not consistent with DGV, indicating that the ground truth might not include all the CNVs. From the first, second and forth column, it is shown that SegSeq, CNVnator and readDepth tend to detect unique CNVs, *i.e.*, detected by only one method. These unique CNVs might be false positives. From the last three columns, CNV-seq and FREEC detect CNVs with high reliability.

**Table 5:** Selected domains of 7-way Venn diagram of CNV called by a single program.

CNV-seq	0	0	0	0	1	1	1
FREEC	0	0	0	0	1	1	1
readDepth	0	0	0	1	1	1	1
CNVnator	0	1	1	0	1	1	1
SegSeq	1	0	0	0	0	1	1
EWT	0	0	1	0	1	1	1
DGV	0	0	0	0	0	0	1
Block number	32007	2293	1235	1339	2961	2705	1507

Each column represents a domain. ‘0/1’ denotes a normal/CNV status.

**Figure 9:**
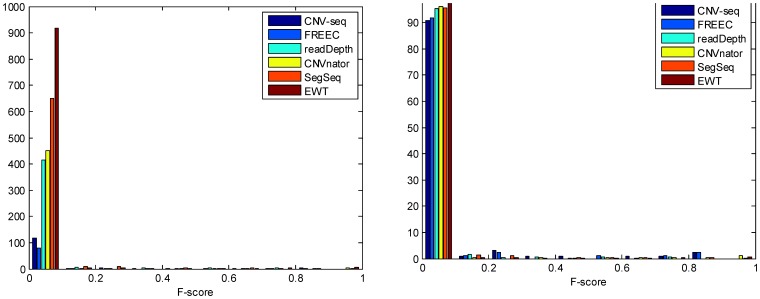
Distribution of *F*-scores of detected CNVs. The left panel represents the number of CNVs, and the right one represents the percentage.

To study the quality of detected CNVs, the *F*-score of each CNV was calculated. The distribution of *F*-score is shown in Figure 9. It is shown that CNV-seq and FREEC detect less (both in number and in percent) CNVs with low quality (*F*-score<0.1) compared with the rest methods. This is consistent with the 7-way Venn diagram analysis: CNV-seq and FREEC are relatively conservative, and only report reliable CNVs.

### Computational Demand

The experimental environment was established in linux openSUSE 11.3, and the desktop computer has a dual-core 2.8 GHz x86 64 bit processor and 6 GB memory.

**Figure 10:**
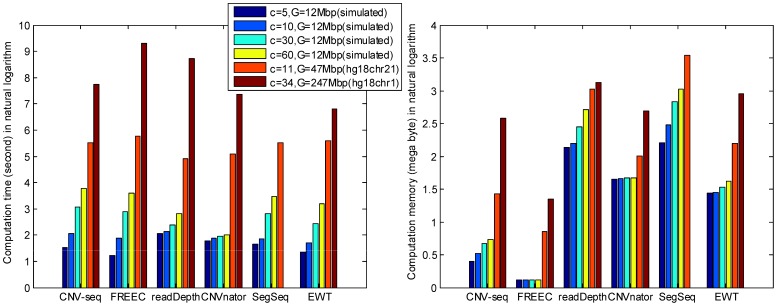
Computational demands. The left panel is the computation time in second, and the right one is the peak memory usage in megabyte.

The computation time and memory usage are shown in Figure 10. It is shown that for small data size (coverage and genome length), computation time of CNV-seq, FREEC, SegSeq and EWT approximately increases linearly with the data size, while CNVnator achieves the best speed because of the very low increase of the computation time. The memory usage of CNVnator and FREEC are almost fixed for small data. For large data size, EWT runs fastest among all the six methods, and FREEC achieves the best memory efficiency among all the methods. Note that the missing bar of SegSeq is caused by ‘out of memory’, so SegSeq is not suitable for large data size when memory is not sufficient.

In summary, in terms of both computation time and peak memory usage, CNV-seq, CNVnator and EWT are computationally efficient compared with other methods; readDepth is fastest for medium size data, and EWT has a good balance between computational complexity and storage.

## Discussion

In this paper, six publicly available CNV detection methods: CNV-seq, FREEC, readDepth, CNVnator, SegSeq and EWT, were compared comprehensively on both simulated and real data. This comparative study provides guidelines for investigators to choose the most appropriate method according to their specific requirements and data set.

The following guidelines are proposed based on our comparative studies (Table 6).

**Table 6:** Guidelines for the tested CNV detection methods.

CNV-seq	good copy number estimation
FREEC	high TPR; low memory usage
readDepth	good break point and copy number estimation; low FPR
CNVnator	good break point and copy number estimation; low FPR
SegSeq	high TPR and FPR
EWT	good break point estimation; high TPR; low FPR; fast computation speed

- readDepth, CNVnator and EWT achieve better break point estimation among all the tested CNV detection methods;
- CNVnator, readDepth and CNV-seq provide better copy number estimation compared with the rest;
- When low FPR is preferable, readDepth, EWT and CNVnator are better choices;
- When high TPR is preferable, FREEC, SegSeq and EWT are better choices;
- If the computation speed/memory usage is the first priority, EWT/FREEC should be used;
- EWT has the best balance between computation time and memory usage.

An interesting finding based on our simulation is that, the single copy gain (*c* = 3) is less easily detected compared with the single copy loss (*c* = 1). As shown in Figure 4, the circle (single copy gain) is lower than the diamond (single copy loss) for all the six methods except SegSeq. Further literature study supported this *in silico* finding. In [26] Klambauer *et al.* gave a theoretical proof, showing that the average read count for copy number 2 has a higher probability to be drawn from a copy number 3 than from a copy number 1 distribution. In other words, CNVs with copy number 3 are more likely to be assigned to copy number 2, yielding lower probability to be detected.

As the developer stated, the LASSO-based segmentation is robust against outliers. From the simulation experiments, it is shown that FREEC is robust to detect CNVs. However, since the L−1 norm used in the LASSO yields bias on the estimates of the amplitude [32], the copy number estimates of FREEC are not accurate, as shown in the overall performance ranking. EWT transfers the read count ratio into Z-score, and tests over an interval of consecutive windows. Since the statistics is well designed, EWT works robustly over all simulation conditions. CNV-seq fails to detect low level copy number deviations (*i.e.* copy number 1 and 3); we found that the designed statistics is not sensitive to this task: From Figure 4, The TPR is only 0.2 and 0.03 respectively with default setting where the *p*-value is 1e−3. When the *p*-value is increased to 1e−2, the TPR improves to 0.65 and 0.25 respectively. readDepth uses CBS to segment the read depth signal, and calls CNVs with the cutoff derived from the negative-binomial model. In this model, an over dispersion parameter, or the variance mean ratio needs to be predefined. When we decreased this parameter to as small as 0.1 from the recommended value 1, the TPR of readDepth at copy number 1 can reach above 0.99; but the TPR at copy number 3 cannot be improved by trying different combinations of parameters. However, at copy number 4, the TPR can easily reach above 0.99 with recommended setting. For CNVnator, the mean-shift algorithm is employed to segment the read depth signal. In the mean-shift algorithm, a local 2D density function has to be estimated to determine the breakpoints. And in the estimation of this density function, the so-called ‘bandwidth for the bin index’ parameter *H_b_*, which is in fact the number of neighboring consecutive non-overlapping bins, needs to be predefined. This *H_b_* and the bin size jointly determine the resolution of CNVnator. In order to have multiple resolutions, *H_b_* is increased from 8 to 128. The default bin size is 100 bp, so the theoretical extreme resolution is 800 bp. This minimal resolution is consistent with Figure 2, in which the TPR is 0 and 0.09 at CNV size 0.8 kbp and 1 kbp, respectively. SegSeq fails at copy number zero. From the experiments, we observed that when the copy number is 0, SegSeq always reports two small sized CNVs respectively at the two boundaries of the ground-true CNVs, but never spans the entire ground-true CNV. We found that the reason is as follows: SegSeq uses the log ratio of the case and control reads count as statistic, so when the copy number is zero, or the case reads count is zero, the log ratio is negative infinity, which might cause further exception in the finite precision personal computer. As a result, the software is unable to report this CNV region.

The above findings are based on the simulation experiments, in which the effect of each factor can be studied orthogonally, providing insight into the differences among the CNV detection methods. The simulations only focus on the main characteristics of CNV (*e.g.* single copy length and copy number). However, due to the complexity of human genomes, there are factors that are not considered in the simulation. *e.g.,*the multiple alignment loci problem and GC-content correction. Because of the existence of SNP, indels, and sequencing error, a read can be aligned to multiple loci. One way is to use only the unique mapped reads to count the read depth signal [14], while the other way is to assign a random locus [23] to the multiple loci aligned read, and then count the read depth signal. Since these two ways have been discussed in [30], [31], [33], the simulations did not consider this multiple loci problem. In our experiments, we use the default setting of Bowtie, which is similar to MAQ's default policy [30], such that the best alignments with fewer mismatches are outputted. If a read has multiple alignments with the same quality score, a random locus is selected. Under this strategy, the methods without a control perform the best [26]. We note that the read length is often greater than 36 bp, which can decrease both mismatches and multiple alignments. Therefore, the read depth signal can be affected by the change of the read length. This improvement is equivalent to multiplying a factor by the read depth signal, and so does not affect the results of the CNV detection methods based on DOC. The GC-content bias, which is caused by the non-uniform sampling, affects the read depth significantly when a control data is absent. Since there is no standard formulation between GC-content and sampling distribution [21], in our simulation, we use the GC-content profile as the sample probability factor, which therefore may not reflect the real characteristics of GC-content deviation. This is the limitation of the simulation experiments.

Since performance varies depending on the parameter settings, the configuration of the parameters follows these rules. 1) The shared parameters are set the same for all the methods. Since the recommended GC-content correction bin size of readDepth and EWT is 100 bp, we also set the bin size of CNVnator to 100 bp. The smallest *H_b_* parameter (number of consecutive bins) of CNVnator is 8, so we also set the ‘filter’ parameter of EWT to 8, yielding the size of the smallest detectable CNV to be 800 bp. Therefore, the window size of FREEC and SegSeq is set to 800 bp. The thresholds for CNV-seq and FREEC are set to 0.6. The *p*-value for CNV-seq, *P_init_* and *P_merge_* for SegSeq, false detection rate for readDepth are all set to 1e−3. 2) The parameter that is specific for each method is tested after the shared parameters are fixed. The ‘step’ parameter of FREEC is set to 400 bp; the ‘overDispersion’, ‘percCNGain’ and ‘percCNLoss’ of readDepth are set to 1, 0.01, and 0.01, respectively; the ‘bigger-window’ and ‘minimum-windows-required’ parameter of CNV-seq are set to 1 and 2 respectively. Note that CNV-seq can calculate the window size adaptively when *p*-value is given. We set this *p*-value to 1e−3 for other experiments except when the single copy length is 0.8 and 1 kbp, where the calculated window size is larger than 1 kb. In these two cases (0.8 and 1 kbp), we set the window size to 0.8 kbp, to agree with other methods.

As each algorithm has its own set of parameters to be tuned for different data, it is difficult to tune the parameters for each data set. Also to consider the configurations described in the previous paragraph, some algorithms may not work at their best performance. This might have effect on the comparative results, which need further study.

As the next-generation sequencing is a promising technology, more robust and powerful CNV detection software are needed to efficiently process the huge amount of short read data efficiently. Existing algorithms can be further improved in several aspects: (1) as each algorithm has its own strength and weakness, existing algorithms can be integrated to achieve highly accurate yet robust estimation. One approach is to combine the PEM with the complementary DOC signature, *e.g.* CNVer and CopySeq, to improve the break point position accuracy. Another approach is to process multiple samples simultaneously, *e.g.* cn.MOPS and JointSLM in the population studies, which can decrease the FPR [26]. (2) There are defects and bugs occasionally in the current software. *e.g.* cnD works for some data set, but occasionally fails for other data set simulated with the same parameters. Therefore further works are needed to improve the robustness of the software. (3) A user more friendly software is needed because some software tools require to configure more than one file, causing inconvenience. To this end, we are in the process of developing new approaches for the detection of CNVs with sparse regression models and the corresponding software [25], which will be reported elsewhere.

## Supporting Information

Figure S1 The precision-recall with different single copy length: 0.8 kbp (triangle), 1 kbp (diamond), 2 kbp (circle) and 6 kbp (square). The coverage is fixed to 5 and copy number is fixed to 6.
(TIF)

Figure S2 The precision-recall with different copy number: 0 (triangle), 1 (diamond), 3 (circle) and 6 (square). The coverage is fixed to 5 and single copy length is fixed to 6 kbp.
(TIF)

Figure S3 The precision-recall with different coverage: 3 (triangle), 5 (diamond), 10 (circle), 30 (square). The copy number is fixed to 6 and single copy length is fixed to 6 kbp.
(TIF)

Table S1 The *F*-scores in the simulation studies.
(DOCX)

Table S2 The *p*-values (pairwise Wilcoxon rank-sum test) of break point position estimation.
(DOCX)

Table S3 The *p*-values (pairwise Wilcoxon rank-sum test) of copy number estimation.
(DOCX)
