# Supplementary figures and images for: Comparative Studies of Copy Number Variation Detection Methods for Next-Generation Sequencing Technologies
Source: PLoS One. 2013 Mar 20;8(3):e59128. doi: 10.1371/journal.pone.0059128 (PMC3604020; doi:10.1371/journal.pone.0059128)

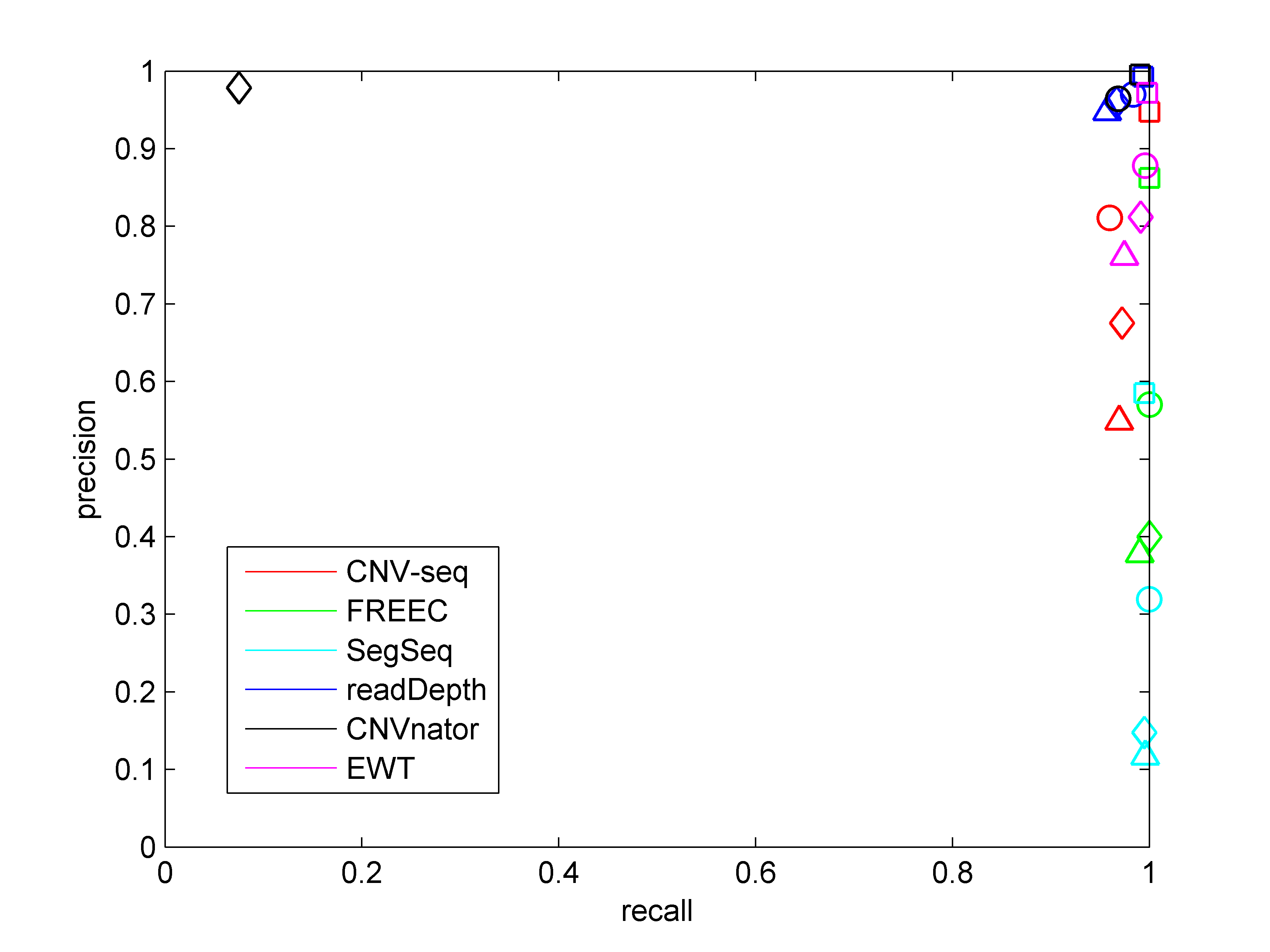

Supplement: Figure S1 — The precision-recall with different single copy length: 0.8 kbp (triangle), 1 kbp (diamond), 2 kbp (circle) and 6 kbp (square). The coverage is fixed to 5 and copy number is fixed to 6. (TIF) [file pone.0059128.s001.tif]

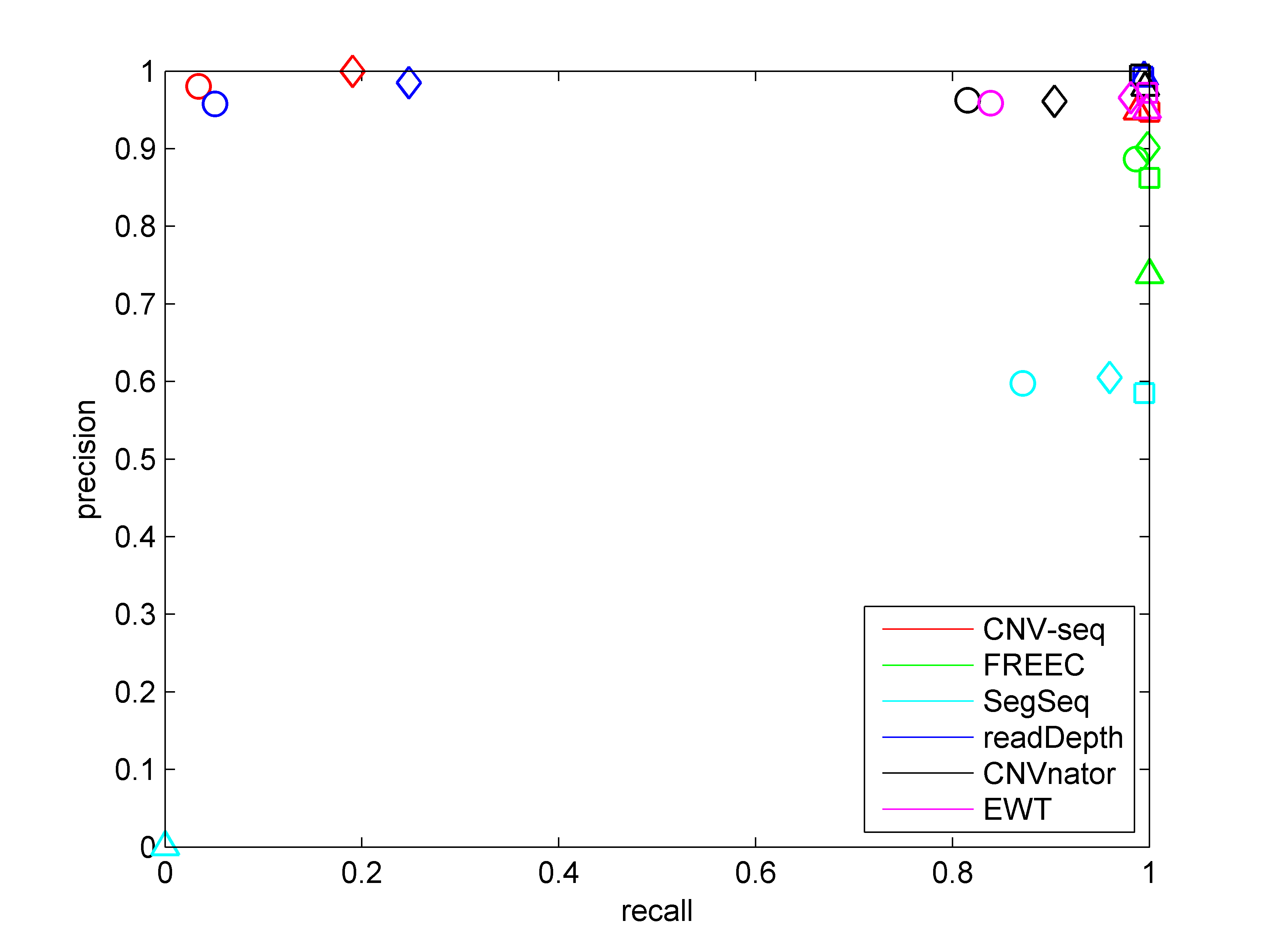

Supplement: Figure S2 — The precision-recall with different copy number: 0 (triangle), 1 (diamond), 3 (circle) and 6 (square). The coverage is fixed to 5 and single copy length is fixed to 6 kbp. (TIF) [file pone.0059128.s002.tif]

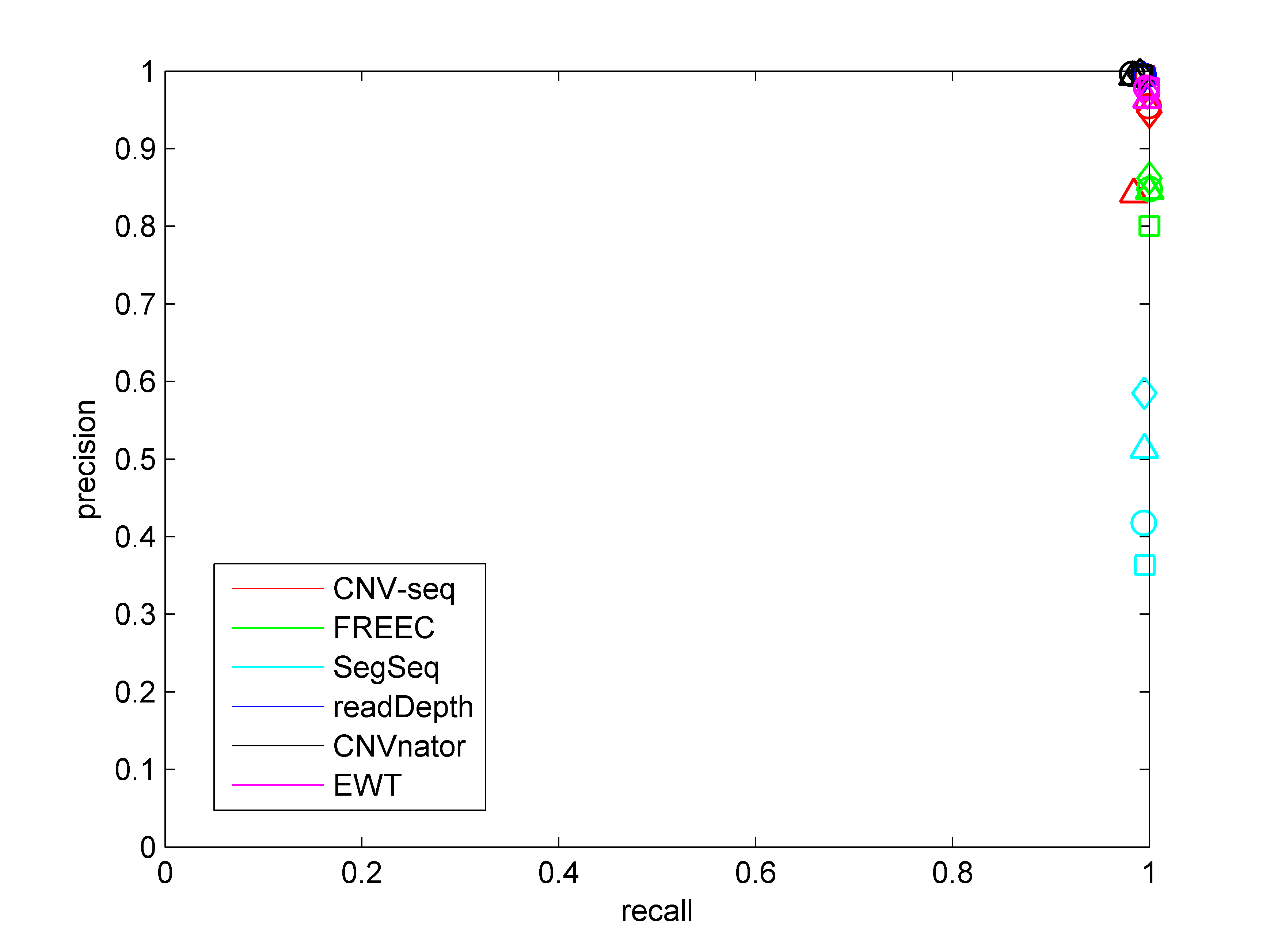

Supplement: Figure S3 — The precision-recall with different coverage: 3 (triangle), 5 (diamond), 10 (circle), 30 (square). The copy number is fixed to 6 and single copy length is fixed to 6 kbp. (TIF) [file pone.0059128.s003.tif]
