# Supplementary material for: Comparative Studies of Copy Number Variation Detection Methods for Next-Generation Sequencing Technologies
Source: PLoS One. 2013 Mar 20;8(3):e59128. doi: 10.1371/journal.pone.0059128 (PMC3604020; doi:10.1371/journal.pone.0059128)
Supplement: Table S1 — The F-scores in the simulation studies. (DOCX) [file pone.0059128.s004.docx]

|  | CNV-seq | FREEC | readDepth | CNVnator | SegSeq | EWT |
| --- | --- | --- | --- | --- | --- | --- |
| *l*=0.8 kbp, *n*=6, *c*=5 | 0.701 | 0.547 | 0.952 | NA | 0.209 | 0.854 |
| *l*=2 kbp, *n*=6, *c*=5 | 0.879 | 0.726 | 0.976 | 0.966 | 0.484 | 0.933 |
| *l*=6 kbp, *n*=6, *c*=5 | 0.973 | 0.925 | 0.993 | 0.993 | 0.736 | 0.984 |
| *l*=6 kbp, *n*=0, *c*=5 | 0.968 | 0.848 | 0.993 | 0.987 | 0 | 0.973 |
| *l*=6 kbp, *n*=1, *c*=5 | 0.320 | 0.947 | 0.395 | 0.931 | 0.742 | 0.973 |
| *l*=6 kbp, *n*=3, *c*=5 | 0.066 | 0.933 | 0.096 | 0.882 | 0.708 | 0.894 |
| *l*=6 kbp, *n*=6, *c*=3 | 0.907 | 0.916 | NA | 0.988 | 0.676 | 0.980 |
| *l*=6 kbp, *n*=6, *c*=10 | 0.976 | 0.917 | 0.994 | 0.989 | 0.588 | 0.987 |
| *l*=6 kbp, *n*=6, *c*=30 | 0.994 | 0.889 | 0.994 | 0.993 | 0.532 | 0.988 |
