# Supplementary material for: Comparative Studies of Copy Number Variation Detection Methods for Next-Generation Sequencing Technologies
Source: PLoS One. 2013 Mar 20;8(3):e59128. doi: 10.1371/journal.pone.0059128 (PMC3604020; doi:10.1371/journal.pone.0059128)
Supplement: Table S2 — The p-values (pairwise Wilcoxon rank-sum test) of break point position estimation. (DOCX) [file pone.0059128.s005.docx]

|  | CNV-seq | FREEC | readDepth | CNVnator | SegSeq | EWT |
| --- | --- | --- | --- | --- | --- | --- |
| CNV-seq |  | 8.52E-36 | 1.07E-42 | 6.26E-40 | 3.95E-05 | 7.25E-34 |
| FREEC |  |  | 3.95E-39 | 8.28E-37 | 3.11E-04 | 3.82E-31 |
| readDepth |  |  |  | 8.78E-06 | 3.93E-06 | 0.9721 |
| CNVnator |  |  |  |  | 6.66E-05 | 3.65E-04 |
| SegSeq |  |  |  |  |  | 1.14E-06 |
