# Supplementary material for: Comparative Studies of Copy Number Variation Detection Methods for Next-Generation Sequencing Technologies
Source: PLoS One. 2013 Mar 20;8(3):e59128. doi: 10.1371/journal.pone.0059128 (PMC3604020; doi:10.1371/journal.pone.0059128)
Supplement: Table S3 — The p-values (pairwise Wilcoxon rank-sum test) of copy number estimation. (DOCX) [file pone.0059128.s006.docx]

|  | CNV-seq | FREEC | readDepth | CNVnator | SegSeq | EWT |
| --- | --- | --- | --- | --- | --- | --- |
| CNV-seq |  | 1.66E-19 | 1.67E-13 | 3.55E-30 | 4.27E-20 | 1.27E-10 |
| FREEC |  |  | 1.34E-22 | 2.91E-30 | 1.02E-04 | 2.74E-09 |
| readDepth |  |  |  | 9.99E-30 | 1.07E-21 | 3.01E-11 |
| CNVnator |  |  |  |  | 1.33E-24 | 5.88E-13 |
| SegSeq |  |  |  |  |  | 2.14E-20 |
